# The relationship between physical and psychosocial workplace exposures and life expectancy free of musculoskeletal and cardiovascular disease in working life – an analysis based on German health insurance data

**DOI:** 10.1186/s12889-024-19721-1

**Published:** 2024-08-13

**Authors:** Lieselotte Mond, Janice Hegewald, Falk Liebers, Jelena Epping, Johannes Beller, Stefanie Sperlich, Jona Theodor Stahmeyer, Juliane Tetzlaff

**Affiliations:** 1 Medical Sociology Unit, Hannover Medical School, Carl-Neuberg Str. 1, 30625 Hanover, Germany; 2 Federal Institute for Occupational Safety and Health (BAuA), Berlin, Germany; 3 AOK Niedersachsen- Statutory Health Insurance of Lower Saxony, Hanover, Germany

**Keywords:** Disease-free life expectancy, Job exposure matrix, Claims Data, Cardiovascular diseases, Musculoskeletal diseases, Physical workplace exposures, Psychosocial Workplace exposures

## Abstract

**Background:**

Against the backdrop of the debate on extending working life, it is important to identify vulnerable occupational groups by analysing inequalities in healthy life years. The aim of the study is to analyse partial life expectancy (age 30–65) [1] free of musculoskeletal diseases (MSD) and [2] free of cardiovascular diseases (CVD) in occupational groups with different levels of physical and psychosocial exposures.

**Methods:**

The study is based on German health insurance claims data from 2015 to 2018. The study population comprises all employed insured persons aged 18 to 65 years (*N* = 1,528,523). Occupational exposures were assessed using a Job Exposure Matrix. Life years free of MSD / CVD and life years with MSD /CVD during working age were estimated using multistate life tables.

**Results:**

We found inequalities in MSD-free and CVD-free life years, with less disease-free years among men and women having jobs with high levels of physical and psychosocial exposures. Men with low physical exposures had 2.4 more MSD-free and 0.7 more CVD-free years than men with high physical exposures. Women with low psychosocial exposures had 1.7 MSD-free and 1.0 CVD-free years more than women with high psychosocial exposures.

**Conclusions:**

Employees in occupations with high physical and psychosocial demands constitute vulnerable groups for reduced life expectancy free of MSD and CVD. Given the inequalities and high numbers of disease-affected life years during working age, the prevention potential of occupational health care and workplace health promotion should be used more extensively.

**Supplementary Information:**

The online version contains supplementary material available at 10.1186/s12889-024-19721-1.

## Introduction

Demographic ageing leads to a decreasing share of contributors to the pension systems in relation to their beneficiaries and is a major challenge to pay-as-you-go pension systems. In this context, many countries have increased retirement age to deal with this change and the advantages and disadvantages of lengthening working life are discussed intensively. Since working adults in daily life spend a large part of the day at work, physical and psychosocial job exposures can have a huge impact on health [1–4]. This impact can even result in labour market exits. Musculoskeletal diseases (MSD) and cardiovascular diseases (CVD) are frequent reasons for incapacity to work [5, 6], early labor market exits [7, 8] and occupy rank 1 and 2 of diseases with the highest number of “Disability-Adjusted Life Years” (DALYS) in Germany [9]. In this study, we calculated life expectancy with and without [1] MSD and [2] CVD for working people with different levels of physical and psychosocial job exposures and job demands. Thus, inequalities and vulnerable occupational groups can be identified.

The number of disease-free life years with regard to different levels of job exposures and demands contributes to the scientific foundation of the ongoing debate on retirement age in Germany. In contrast to estimates of simple disease risks, it provides information on the estimated duration of life with or without a specific disease. This study focuses on specific diseases rather than on more general health outcomes for which the derivation of prevention needs is ambiguous. There are only a few studies on disease-free life expectancy, both internationally [10, 11] and for the German population [12, 13]. They show that disease-free LE follows a gradient in terms of income, life style or other factors. To our knowledge, there is so far no study that depicts life expectancy free of MSD or CVD for the working population and no study that analysed inequalities in disease-free life expectancy for different levels of job exposures in Germany.

Occupational exposures and demands such as chemical exposures, high physical workload, long work hours, and psychosocial exposures are important risk factors for many chronic diseases [1, 14, 15]. Job exposure matrices (JEM) have been frequently used for estimating exposure to work hazards. Exposures were estimated on the basis of job titles, industry information or population exposure data and were used to study the relationship between occupational exposures (and workloads) and various health outcomes [16–18], including MSD and CVD [2, 3, 19]. The association between physical and psychosocial exposures at work and diseases (including MSD and CVD) is evident: Studies frequently reported a clear effect of both physical and psychosocial job exposures on the risk of MSD [2, 20–23] and CVD [4, 24–29]. This underlines the importance of studies on inequalities in the disease-free life span among the working population. Based on health insurance data this study addresses this topic by answering the following questions:

In working adults insured by the AOK Lower Saxony (Germany),

- do people with high *physical* or high *psychosocial* job exposures have lower life expectancy free of *musculoskeletal* diseases (and higher life expectancy with musculoskeletal diseases) than people with intermediate or low physical or psychosocial job exposures?
- do people with high *physical* or high *psychosocial* job exposures have lower life expectancy free of *cardiovascular* diseases (and lower life expectancy with cardiovascular diseases) than people with intermediate or low physical or psychosocial job exposures?
- are there differences between men and women with respect to the inequalities analysed above?

## Methods

### Data

This observational study is based on longitudinal routine health insurance data from the AOK Niedersachsen (AOKN) covering the years 2015 to 2018. We pooled the data from 2015 to 2018 and analysed the research questions in a prospective study design.

AOKN is one of the biggest German statutory health insurance providers and covers about 37% of the inhabitants of Lower Saxony (German: Niedersachsen). Health insurance is mandatory in Germany, and only people earning above a certain income threshold (66,600 Euro gross income in 2023), the self-employed and civil servants are allowed to switch to private health insurance. A broad range of health services (including all necessary and evidenced-based health services) is covered by the statutory health insurance in Germany. The study population comprises all employed AOKN insured persons aged 18 to 65 years. The data are anonymous and their use for scientific purposes is regulated by federal law. Consent to participate or ethical approval is therefore not required. All methods were carried out according to the relevant guidelines.

### MSD and CVD onset

The analysed key variables are incident cardiovascular diseases (CVD) and each new occurrence (including recurrences) of musculoskeletal diseases (MSD) leading to a health service claim (e.g. sick leave cases, treatment or monitoring). The diseases were coded according to ICD-10 GM, and were documented by the physicians. CVD includes chronic heart disease, heart failure, angina pectoris, peripheral arterial occlusive disease as well as myocardial infarction and stroke (see Table 1). MSD includes all “diseases of the musculoskeletal system and connective tissue” (see Table 1). For myocardial infarction and stroke (I21-22 and I60-64), only inpatient cases with the infarction or stroke as a principal diagnosis were considered; for all other CVD and all MSD, coded diagnoses from the inpatient and outpatient sectors were used. To increase the validity of the coded diagnoses, the established “M2Q-criterion” was applied [30]. This means that a diagnosis from the outpatient sector is only counted if it is confirmed by another coding (outpatient or inpatient) in another quarter of the year in question. Since disease onset (i.e. new cases) was the endpoint of interest, at least one year free of disease had to precede the diagnosis to differentiate between incident and prevalent cases. Prevalent cases were excluded from the study population. This procedure was already methodically elaborated in previous studies [31].

For MSD, occurrence and recurrence are considered. A person with previous MSD-diagnosis is defined as “recovered” if he or she had no coded MSD-diagnosis for at least one year, implying that there was no longer a need for the use of health care services. In contrast to CVD, some of the most prevalent MSD (e.g. back pain, muscle pain) are acute and may be reversible.

**Table 1:** ICD 10 – codes for Musculoskeletal and Cardiovascular diseases

**Musculoskeletal diseases (MSD)**	
Arthropathies	M00-25
Systemic connective tissue disorders	M30-36
Dorsopathies	M40-54
Soft tissue disorders	M60-79
Osteopathies and chondropathies	M80-94
Other disorders of the musculoskeletal system and connective tissue	M95-99
**Cardiovascular Diseases (CVD)**	
Ischaemic heart diseases	I20-25
Heart failure	I50
Cerebrovascular diseases	I60-64
Artherosclerosis of arteries of extremities and Peripheral vascular disease, unspecified	I70.2 and I73.9

### Occupational exposures

JEM was used to estimate physical exposures (including workload) and psychosocial occupational conditions. The JEM used in this study [32, 33] has the advantage of being available for all occupations coded according to the official German classification of occupations KldB (“Klassifikation der Berufe” - Classification of Occupations). As KldB is also recorded in the statutory health insurance data (for all employees who are subject to compulsory statutory health insurance coverage), this JEM can be merged with the routine data of the AOKN. The JEM is based on the *2006/2012 BiBB/BAuA Employment Survey* conducted by the Federal Institute for Vocational Education and Training (BIBB) and the Federal Institute for Occupational Safety and Health (BAuA) in 2006 and 2012, using a representative sample of 20,000 German employees aged 15 to 65 [34]. A total of 39 items from the survey were used as indicators of physical exposure, including workload (e.g. frequent standing or frequent working in smoke, dust or under gases, vapours) or psychosocial exposure (e.g. frequently having to work to the limit of one´s ability, never receiving support from colleagues or shift work). Further details on the included items and operationalization of the JEM can be found in the original publication by Kroll [32].

The validity of the JEM based on the KldB could be shown on the basis of health indicators in the 2006 employment survey as well as on the basis of health indicators from the 2009 GEDA Study [32, 33]. We are not aware of a more recent JEM that would match the health insurance data and is similarly well validated.

Employers are obliged to report information on the occupation of every employee to the health insurance fund using the KldB. Persons with missing information on their occupation had to be excluded from the analysis (below 1%). Kroll´s “Job Exposure Matrix (JEM) for KldB” [32] was linked to the AOKN data. After merging, each person was assigned two values ranging from 1 (lowest) to 10 (highest), representing the level of physical and psychosocial occupational exposures attributed to their occupation (longest job title within one year). Finally, the 10-item scales were combined into three groups: 1 to 3 (low exposure), 4 to 7 (intermediate exposure) and 8 to 10 (high exposure).

### Statistical analysis

**Fig. 1:**
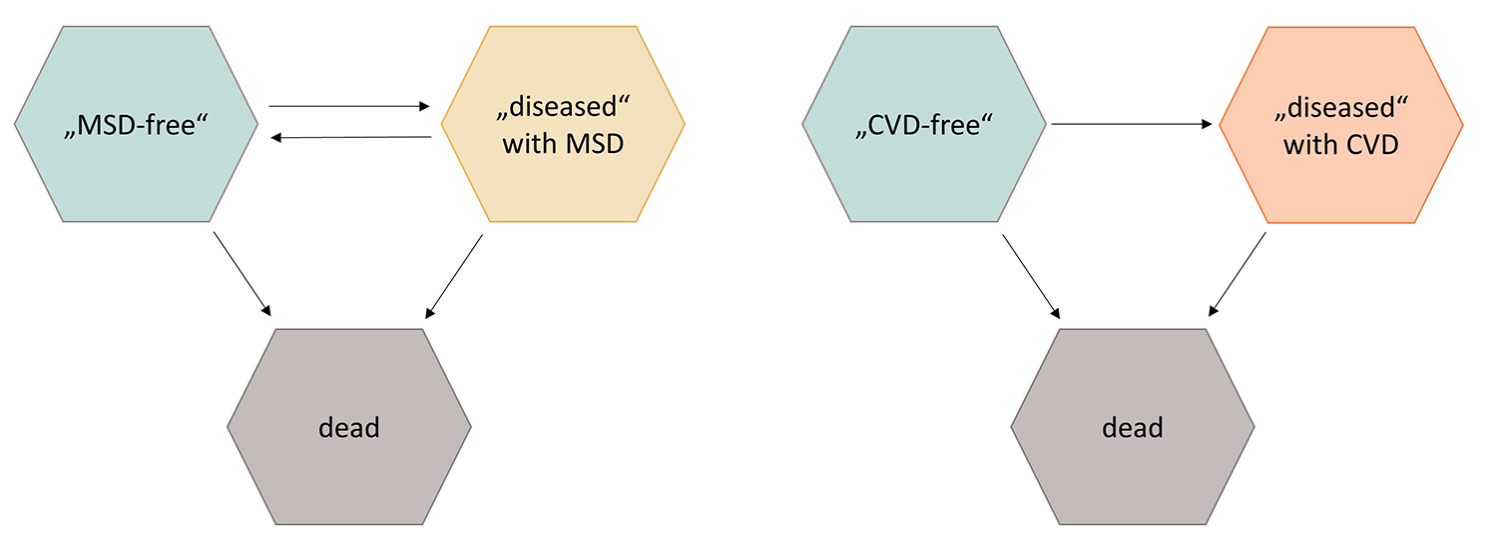
Transition model between the three states: “MSD- / CVD-free” (no coded diagnose), “diseased” (with coded diagnose) and “dead”

Two disease-free partial life expectancies (LE) were considered: the number of years free of MSD (MSD-free LE) and the number of years free of CVD (CVD-free LE) during working life. In addition, the number of years with MSD / CVD were analysed. MSD- / CVD-free LE and LE with MSD / CVD were calculated using multistate life table analysis (MSLT) taking into account the following age-specific transition rates between states: “MSD- / CVD-free” (no coded diagnosis of MSD / CVD, implying no current need for health care services), “diseased” (MSD / CVD prevalent) and “dead” (Fig. 1).

For MSD, re-transition from a state with prevalent MSD to a MSD-free state is possible. The multistate life table analyses was carried out analogously to the increment-decrement life table method according to Palloni [35] and is based on the observed age-specific transition rates between the status shown in Fig. 1. In this way, the number of life years free of disease that can be expected during working life under the transition rates applicable at the time under consideration is determined. 95% CIs were calculated on the basis of 1000 bootstraps. For the analysis, the statistic software Stata 16.0 MP and R (Version 1.1.453) were used.

All expectancies are reported at the age of 30. To cover a broad range of working age, the upper limit is set to the age of 65 years, meaning that partial expectancies at age 30 up to age 65 are reported. Thus, the number of years without disease plus the years with disease add up to 36 years at maximum. Remaining differences to 36 years are due to death before age 66. The analyses were restricted to the age of 65, although many of the considered diseases continue to affect people´s lives far beyond that age. However, in this study the focus was set on the working life to address the question of raising retirement age and to focus on a setting with high potential for prevention. The age of 30 was chosen as an example because this is the age at which most people, even those with long periods of education, should have entered the labour market. Moreover, partial MSD- / CVD-free LE up to age 65 are also reported for all ages (20 to 65) in the appendix (Additional File 1).

## Results

Descriptive statistics of the study population are shown in Table 2. The study populations for the two disease groups differ, as more prevalent cases with MSE during the pre-observation period were excluded than with CVD. The crude occurrence rates for MSE (i.e. first and recurrent diagnoses) and for CVD (i.e. first diagnoses only) tended to be much higher among persons working in fields with higher physical workload and detrimental psychosocial working conditions.

**Table 2:** Descriptive statistic of the study population: person years at risk and crude occurrence rates by exposure group and gender

		Low physical exposure			Intermediate physical exposure			High physical exposure	
		Men	Women		Men	Women		Men	Women
MSE	Person years at risk	87,226	151,673		212,700	310,796		583,119	166,211
	crude occurence rates per 10,000 person years	1,113	1,489		1,356	1,786		1,593	1,929
CVD	Person years at risk	107,429	220,674		275,464	476,152		786,033	260,111
	crude occurence rates per 10,000 person years	82	40		100	60		125	92
		Low psychosocial exposure			Intermediate psychosocial exposure			High psychosocial exposure	
		Men	Women		Men	Women		Men	Women
MSE	Person years at risk	196,547	156,318		301,331	166,208		385,167	306,154
	crude occurence rates per 10,000 person years	1,363	1,519		1,530	1,736		1,520	1,879
CVD	Person years at risk	253,771	229,822		413,899	254,833		501,257	472,281
	crude occurence rates per 10,000 person years	101	42		111	54		125	79

**Fig. 2:**
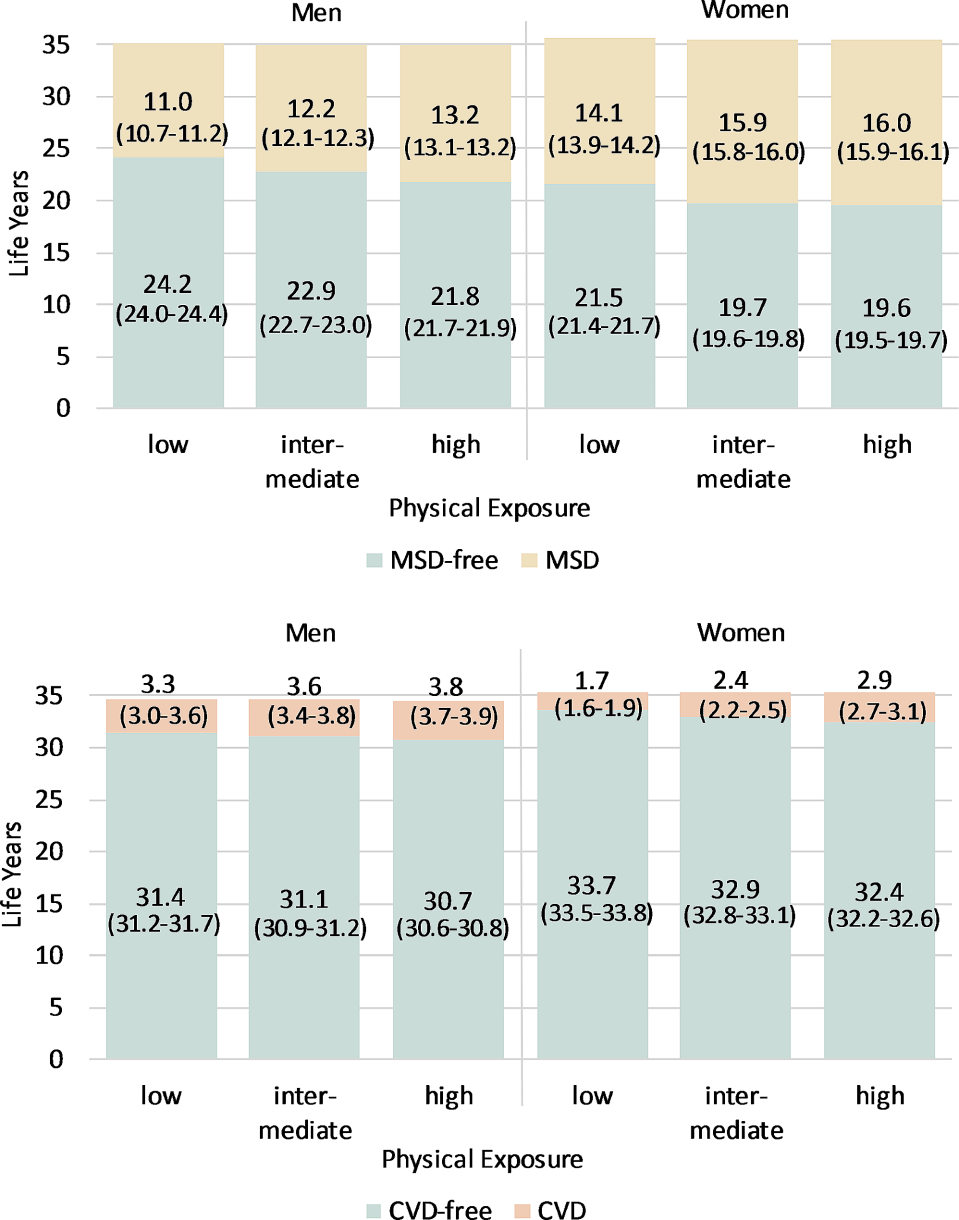
MSD- and CVD-free life expectancy and life expectancy with MSD / CVD at age 30 up to age 65 in occupational groups with different levels of physical exposure with 95% confidence intervals

**Fig. 3:**
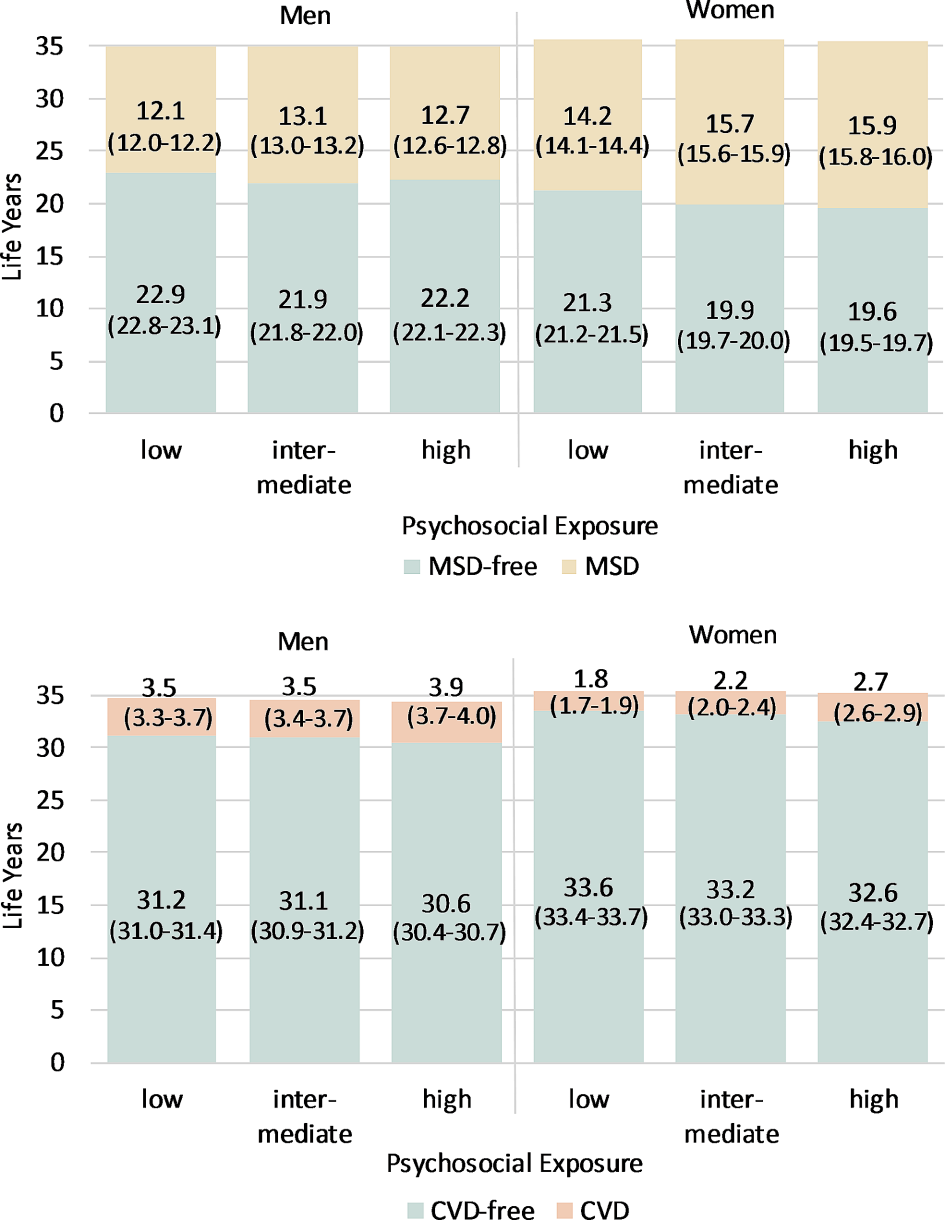
MSD- and CVD-free life expectancy and life expectancy with MSD / CVD at age 30 up to age 65 in occupational groups with different levels of psychosocial exposure with 95% confidence intervals

Figures 2 and 3 show the expected years of life without and with MSD or CVD up to the age of 65 in the different exposure groups at age 30. The inequalities between the exposure groups and genders are present across the entire age range, whereby the absolute differences become smaller with increasing age as the remaining lifespan up to the age of 65 becomes shorter (see Additional File 1). First, MSD-free LE is significantly lower in all groups than the life expectancy free of CVD due to lower occurrence rates of CVD, while life years spent with disease are much higher with respect to MSD than CVD. Furthermore, men can expect to live more years free of MSDs than women. Differences in the total height of the bars are due to different mortality rates in the groups.

Clear gradients between low to high exposed groups were found for both genders and disease groups (Fig. 2). The higher the physical exposure, the fewer the expected life years free of MSD or CVD and the more years spent with the disease. This gradient is most apparent for men with regard to MSD: Here, disease-free LE decreases by more than one year from one exposure group to the next (from 24.2 years for those with low physical exposures to 21.8 years for those with high physical exposures) while years with MSD clearly increase with growing exposure. In most cases, the differences between the exposure groups are statistically significant, which is indicated by non-overlapping 95% CIs (see brackets in Fig. 2).

A similar gradient emerged with respect to psychosocial exposures (Fig. 3): the higher the psychosocial occupational exposure, the lower the number of expected life years free of MSD and CVD and the higher the number of years lived with the disease. The only exception were men with regard to LE free of MSD as we found the lowest MSD-free LE in medium-exposed men (21.9, 95%-CI: 21.8–22.0). The strongest effects can be seen in women with regard to LE with and without MSD: Women working in fields with less harmful psychosocial working conditions can expect to live 1.7 years longer free of MSD (21.3, 95%-CI: 21.2–21.5) than women with high exposures (19.6, 95%-CI: 19.5–19.7). Overall, the gradients across the psychosocial exposure groups are less pronounced than across the physical exposure groups.

## Discussion

### Main findings

This is the first study to analyse disease-free LE for different levels of occupational exposures for two of the most important disease groups in occupational health: MSD and CVD. We distinguished between physical and psychosocial exposures and found inequalities in MSD-free and CVD-free life years during working age, with less disease-free and more disease-affected life years among higher exposed men and women. Inequalities in the MSD-free LE were greater than in CVD-free LE, with higher overall levels of life years without CVD than without MSD. The effect of physical exposures on the two disease groups appeared to be somewhat stronger than the effect of psychosocial exposures, with a difference of 2.2 years in MSD-free LE between men with high and low physical exposure. Psychosocial exposure had a stronger effect on women (Δ1.7 years in MSD-free LE between exposure groups) than on men, who were more affected by physical exposure than women.

### Main findings in the light of previous research

The results of this study are consistent with previous studies showing the impacts of physical and psychosocial exposures on the risk of MSD [2, 20, 21, 23] and CVD [4, 24–29]. A previous study also reported differences between physical exposure groups with regard to health expectancies (i.e. without any chronic illness) [10]. The link between physical job exposures and MSD-free LE shown in this study is, therefore, in Iine with our expectations. Given the broad literature on job strain and increased CVD risk [4, 26, 36], an even stronger association between psychosocial job exposures and CVD-free LE could have been expected (see also “Strengths and limitations”). However, we are not aware of any studies that analysed health expectancies by physical and psychosocial job exposure groups using MSD / CVD as health outcomes, which is why no direct comparisons with previous studies (neither from Germany nor internationally) can be made at the current time.

### Strengths and limitations

The study is based on a very big dataset, which represents a complete population of all insured persons of the health insurance provider. This dataset is representative for Germany in terms of age and gender distributions, while individuals with lower occupational positions are overrepresented [37]. This overrepresentation is taken into account since all analyses are stratified by occupation-related job exposures. In addition, the dataset contains both health and mortality data and is therefore suitable for producing consistent transition rates between different health states for the multistate life table analysis. However, the data do not contain information on cases of myocardial infarction or stroke in which the patient dies before arriving at the hospital. This may lead to an underestimation of inequalities in CVD-free LE between occupational groups, since such cases may occur more often among people of lower SES, utilizing fewer services or living in underserved areas who may be more often affected by high job exposures. To our best knowledge, there are no German studies quantifying inequalities in death after myocardial infarction or stroke before arriving at the hospital. On the other hand, the use of diagnoses coded by physicians has the advantage of being free of any response bias.

As multistate life table analysis is based on incident rates, persons with CVD / MSD in 2015 had to be excluded as prevalent cases. This might lead to selection bias as it may remove more vulnerable unskilled workers without vocational training that are more likely to suffer early in their work life from chronic MSD. However, the multistate life table analysis is considered the preferred method in analysis of health expectancies if incidence and recovery rates change over time [38, 39]. MSLT most accurately reflects the impact of current conditions rather than prevalence rates that reflect the development from the past.

Although JEM neither account for the exposure on the individual level nor for exposure variation in the same job, they provide an inexpensive and useful exposure assessment method for general population studies and can be merged to existing data [3, 40]. However, psychosocial job exposure could vary greatly between individual workplaces, while physical workload seems more homogeneously distributed across jobs within the same group [41]. In this study, findings on the psychosocial exposures and CVD-free LE were weaker than expected, which might be due to an over-generalization of psychosocial working conditions by the JEM. In addition, we have to consider the healthy-worker effect, which can operate in different directions. On the one hand, physically demanding jobs are likely entered by mostly relatively healthy people. On the other hand, people with poor health (e.g. with psychological problems) could have left the employment (and thus our study population), and this may be more common in the groups with higher work exposure levels, through which inequalities could be somewhat underestimated. A migration of workers from high to less demanding jobs due to illness can also be taken into account. However, the level of education and the disease itself may limit the promotion in less stressful jobs. Other factors, such as individual health behaviour can also have a hidden influence.

### Future perspectives

The calculated years of life with and without MSD / CVD during working life are based on transition rates observed in the years 2016–2018. If external conditions (i.e. working conditions, retirement age etc.) change, the number of expected life years with and without disease may change, too. For example, due to the increase in the retirement age, it is to be expected that the number of working years with diseases will increase as well. These increases might be stronger for employees in occupations with high physical and psychosocial exposures, as they may have to work even longer under conditions that are detrimental to their health.

Preventive approaches are available for employees with high physical demands via ongoing occupational health care in Germany, but not systematically for employees with high psychosocial demands, which is currently not a reason for preventive care of individuals. Preventive measures are done on an aggregate (workplace) level, which is anchored in the GDA (Gemeinsame Deutsche Arbeitsschutzstrategie - Joint German Occupational Health and Safety Strategy) [42], but should more focus on individuals rather than on work environment only.

Both the statutory pension insurance schemes and the statutory accident insurance schemes offer individual prevention programs and rehabilitation measures to help people stay in work [43–45]. The statutory health insurance funds also offer support via their prevention programs for both individuals and companies / employers [46, 47]. Given the inequalities in the disease-free life span and the high numbers of years of illness during working age, the prevention potential of existing occupational health care and workplace health promotion should be used more intensively, and prevention focusing especially on reducing the harmful effect of psychosocial work exposures should be established.

Overall, the secondary data from the statutory health insurance funds offer great potential for analysing the links between occupational exposures and the occurrence of illness in the course of employment histories. The potential of these kinds of data in combination with JEM measures should be used in more depth and with respect to a broader range of diseases relevant to health at working age in further research.

## Conclusions

The study investigated for the first time inequalities in disease-free life years among working people with different levels of physical and psychosocial job exposures, focusing on two diseases relevant to population health: CVD and MSD. The study thus emphasizes the great need for effective preventive measures to maintain the health and ability to work of people in occupations with higher job exposure-levels.

### Electronic supplementary material

Below is the link to the electronic supplementary material.

Supplementary Material 1

## Data Availability

The data analysed in this study cannot be made publicly available due to protection of data privacy of the insured individuals by the AOK Niedersachsen (AOKN-Statutory Local Health Insurance of Lower Saxony). The data underlying this study belong to the AOKN. Researchers interested in the data supporting the conclusions of this article can send data access requests to the AOK Niedersachsen using the following e-mail address: AOK.Service@nds.aok.de.

## Abbreviations

AOKN: Statutory health insurance provider in Lower Saxony Germany
BAuA: Federal Institute for Occupational Safety and Health
BIBB: Federal Institute for Vocational Education and Training
CVD: Cardiovascular diseases
CVD-free LE: Life expectancy free of musculoskeletal diseases
DALYS: Disability-Adjusted Life Years
GDA: Joint German Occupational Health and Safety Strategy
ICD-10 GM: International Statistical Classification of Diseases and Related Health Problems, 10th Revision, German Modification
JEM: Job Exposure Matrix
KldB: Classification of Occupations
LE: life expectancy
MSD: Musculoskeletal diseases
MSD-free LE: life expectancy free of musculoskeletal diseases
MSLT: multistate life table analysis
