# Supplementary material for: The relationship between physical and psychosocial workplace exposures and life expectancy free of musculoskeletal and cardiovascular disease in working life – an analysis based on German health insurance data
Source: BMC Public Health. 2024 Aug 13;24:2198. doi: 10.1186/s12889-024-19721-1 (PMC11321087; doi:10.1186/s12889-024-19721-1)
Supplement: Supplementary file 1 — Supplementary Material 1 [file 12889_2024_19721_MOESM1_ESM.docx]

Additional File 1


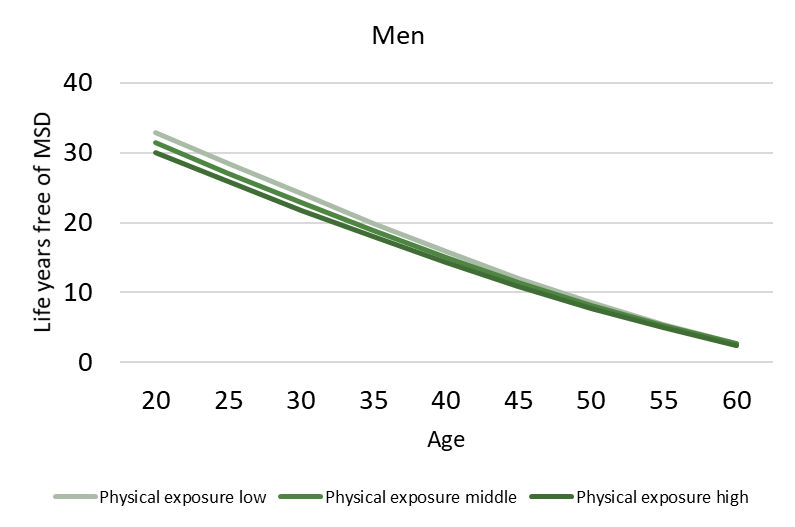

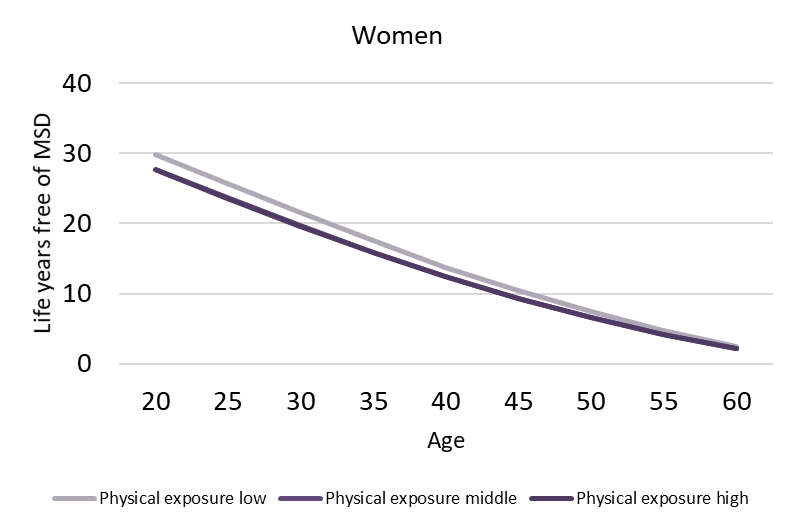


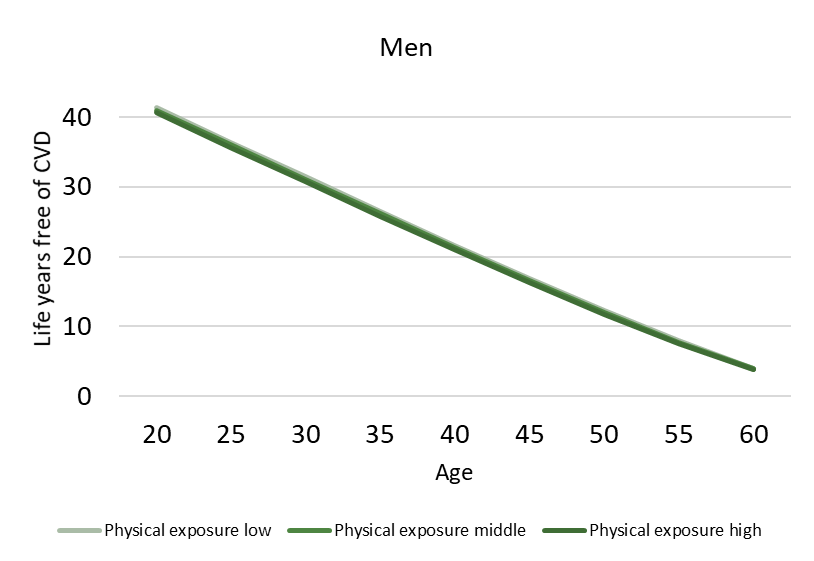

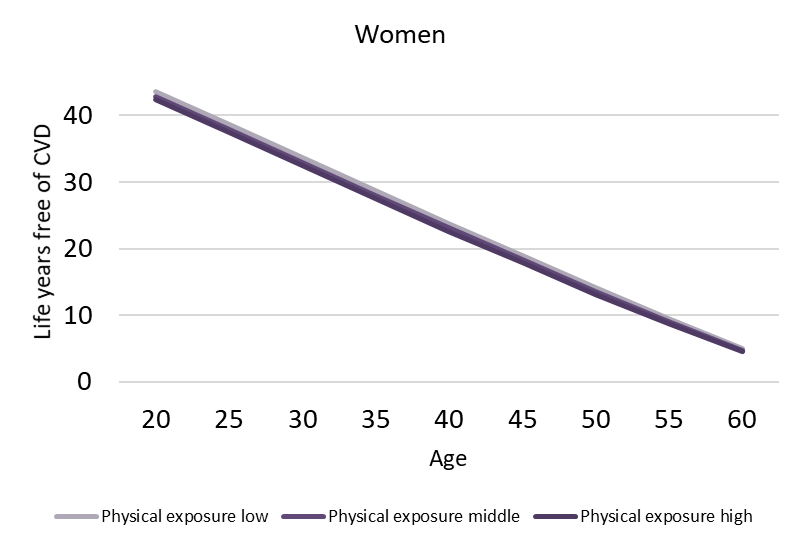


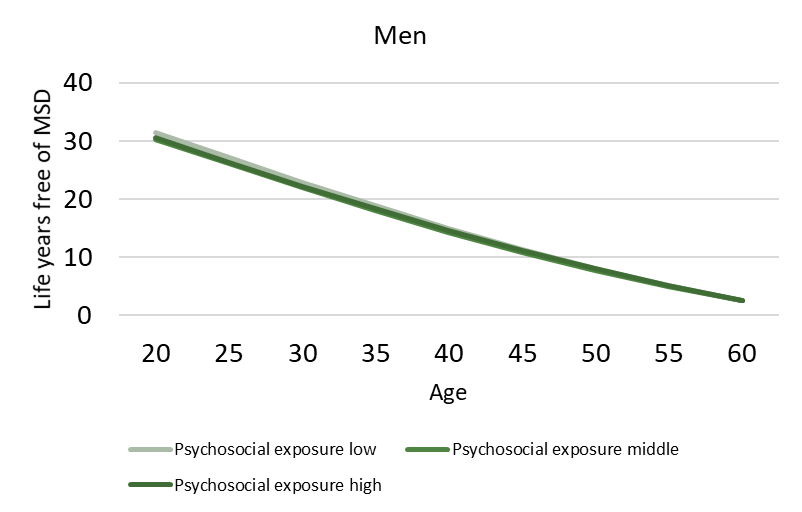

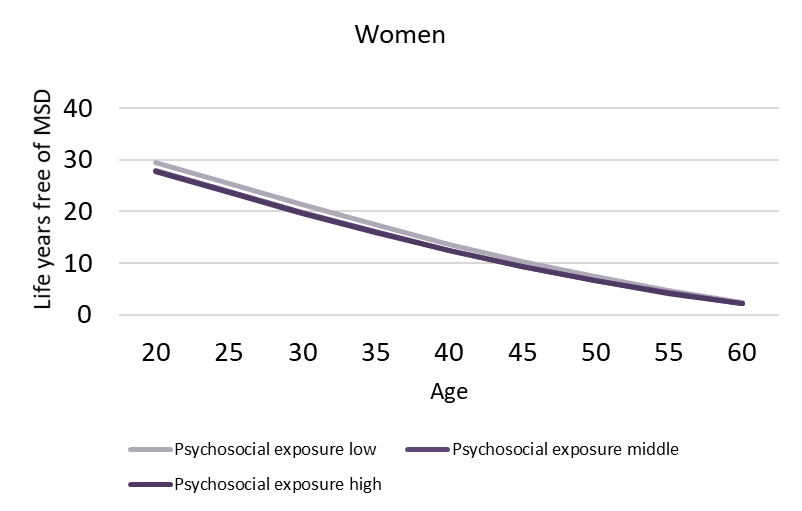

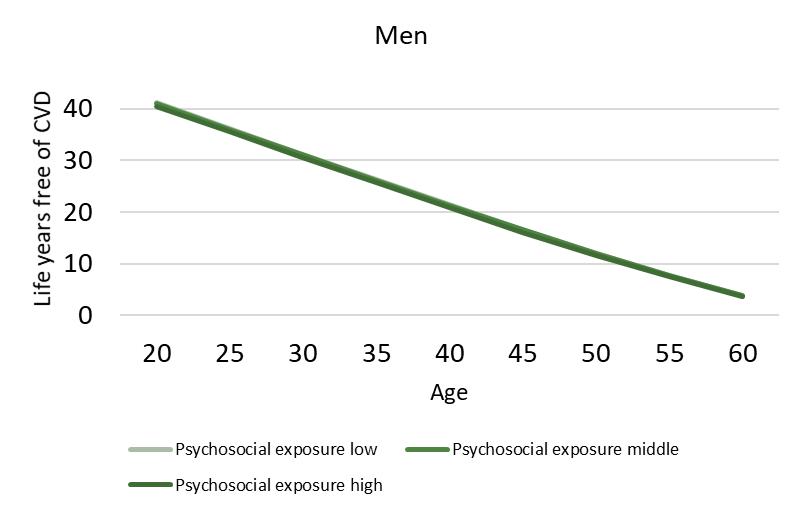

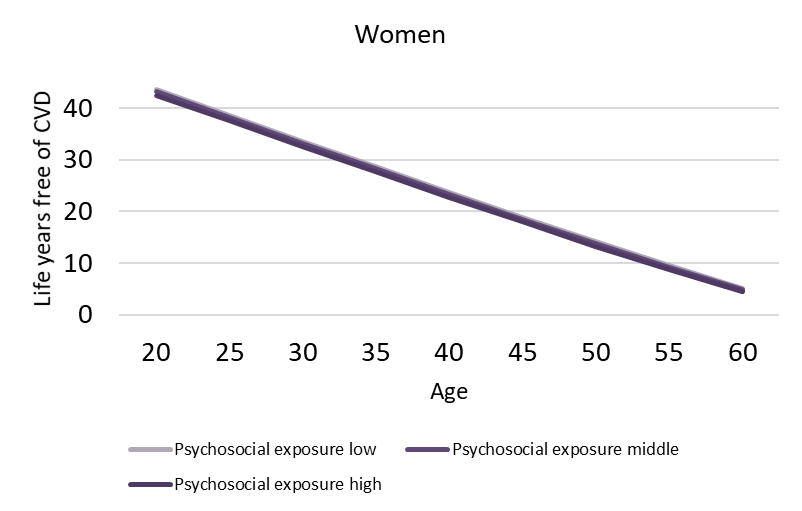


Additional File 1: MSD- and CVD-free life years at ages 20-60 up to age 65 in occupational groups with different levels of physical exposure
